# Drug Survival, Safety, and Effectiveness of Secukinumab for up to 5 Years in Patients with Psoriasis and Psoriatic Arthritis: A Long-Term Real-Life Experience

**DOI:** 10.3390/jpm14070718

**Published:** 2024-07-03

**Authors:** Luca Mastorino, Paolo Dapavo, Caterina Cariti, Sara Susca, Niccolò Siliquini, Michela Ortoncelli, Elena Stroppiana, Anna Verrone, Isotta Giunipero di Corteranzo, Francesco Leo, Pietro Quaglino, Simone Ribero

**Affiliations:** Department of Medical Sciences, Section of Dermatology, University of Turin, 10126 Turin, Italy; pdapavo@cittadellasalute.to.it (P.D.); caterina.cariti@gmail.com (C.C.); ssusca@cittadellasalute.to.it (S.S.); nsiliquini@cittadellasalute.to.it (N.S.); mortoncelli@cittadellasalute.to.it (M.O.); estroppiana@cittadellasalute.to.it (E.S.); averrone@cittadellasalute.to.it (A.V.); isotta.giuniperodicorteranzo@unito.it (I.G.d.C.); francesco.leo144@edu.unito.it (F.L.); pietro.quaglino@unito.it (P.Q.); simone.ribero@unito.it (S.R.)

**Keywords:** psoriasis, secukinumab, PASI, effectiveness, 5 years, long term

## Abstract

Introduction: the selective IL-17 inhibitor secukinumab has demonstrated efficacy and safety in the treatment of moderate–severe psoriasis in recent years. Objective: evaluate effectiveness and drug survival (DS) of secukinumab in patients with psoriasis for up to 5 years. Methods: This is a retrospective study on a monocentric cohort of patients with psoriasis on secukinumab evaluating the achievement of PASI100, PASI90, and PASI ≤ 3 and DS analysis up to 260 weeks. DS multivariate analysis was carried out considering sex, age, age of onset of the disease, obesity, cardiovascular comorbidities, diabetes, involvement of difficult-to-treat sites, psoriatic arthritis, treatment-naïve status, and mean baseline PASI. Results: At baseline, we evaluated 255 patients on secukinumab. PASI100 was reached by 41.7% and 70.6% of patients at weeks 16 and 260, respectively. PASI90 showed a similar trend with 46.5% of patients achieving it at week 16 and 88.2% at week 260. Non-obese patients showed a faster response than patients with obesity in achieving PASI100, PASI90, and PASI ≤ 3, with significant differences at 28 weeks [55% vs. 40% (*p* = 0.033), 64% vs. 49% (*p* = 0.038), and 76% vs. 62% (*p* = 0.036), respectively]. The estimated DS for secukinumab was 84.3% at 12 and 48% at 60 months. Obesity and smoking habits were associated with a higher risk of discontinuation in multivariate models (HR 1.6 CI 1.05–2.45, *p* = 0.028; HR 1.48 CI 1.01–2.17, *p* = 0.043, respectively). Conclusions: Secukinumab showed effectiveness for up to 5 years of treatment, with a high DS and achievement of PASI100, PASI90, and PASI < 3 at these time points. Only obesity reduced the response and maintenance of DS.

## 1. Introduction

Psoriasis is an inflammatory skin disease [1]. The interleukin (IL)23/IL-17 axis and Th17 cells play a fundamental role in its development, and in the last few years, biological targeted therapies focused on its blockade have become available [2,3]. IL-17 is a major player in the development of psoriasis and associated diseases such as peripheral psoriatic arthritis (PsA) and vascular comorbidities [4]. However, recent studies have highlighted the major role of IL-23 in the modulation of the Th17 response and the greater or lesser presence of resident memory T cells in the skin, which are responsible for the persistence and recurrence of psoriatic skin lesions [5]. In contrast, some authors have hypothesized that early psoriasis may have differences from persistent and resistant disease, with a central role for IL-17+ T cells in the selective inhibition of IL-17 in these early forms [6].

Secukinumab, an IL-17A inhibitor approved for the treatment of moderate-to-severe plaque psoriasis and PsA, has shown high levels of clinical efficacy and a favorable safety profile in both short- and long-term clinical trials [7,8]. 

Regarding PsA, it is characterized by a more complex management than PsO, with multiple clinical and laboratory domains to take into account. It is also affected by protean clinical variants and comorbidities, including enthesitis, dactylitis, axial-, nail-, eye-, and bowel-involvement. IL17 inhibitors, such as secukinumab, are effective in several dimensions of the disease, particularly in the skin; however, they fail in the management of eye involvement, and worsen the bowel picture. Real-world evidence of secukinumab in this particular population is even scarcer than in PsO patients [9].

The efficacy of secukinumab has also been assessed on the erythrodermic and pustular forms of psoriasis in recent real-life experience, as in difficult-to-treat regions such as palmoplantar sites [10,11,12]. Real-life studies have highlighted the good efficacy and safety of the drug for up to 2 years of treatment, with conflicting results on drug survival (DS), ranging from 74.3% to 59% [13,14,15,16,17,18,19,20,21,22].

To date, the real-world data effectiveness, safety, and DS of secukinumab at times longer than two years of treatment are scarce [13,17,18]. Only the ENHANCE study by Nguyen et al. reported a persistence of 53.6% at 36 months and 34.2 at 48 months with 53.8% of patients achieving a Psoriasis Area Severity Index (PASI) of 100 and 92.3% achieving PASI75.

Associated factors for the possible discontinuation of therapy vary among studies, and sometimes discordantly, include obesity, female gender, joint involvement, previous biological therapy, short duration of psoriasis (<5 years), and absence of concomitant therapy [13,14,16,18,22].

Given these considerations, we performed a retrospective real-world analysis to investigate the long-term response of secukinumab in patients with psoriasis.

## 2. Methods

This was a monocentric non-interventional observational retrospective study performed at the Dermatologic Clinic of the Turin University Hospital from 1 January 2017 to 1 February 2023 on patients with psoriasis on treatment with secukinumab.

### 2.1. Objectives

We evaluated the achievement of PASI 100, PASI90, and PASI ≤ 3 at 16, 28, 52, 78, 104, 156, 208, and 260 weeks on observed cases. We then performed a comparative evaluation between bio-naïve and bio-experienced patients, obese and non-obese, patients with and without difficult-site involvement (nails, palms and soles, scalp, folds, genitals), and patients with and without PsA, for each outcome at each time point.

DS multivariate analysis was performed using a multivariate model to identify possible predictors of interruption including sex, age, age of onset of disease, obesity, cardiovascular comorbidities, diabetes, involvement of difficult-to-treat sites, PsA, treatment-naïve status, and mean baseline PASI.

### 2.2. Patients

All patients with an age greater than or equal to 18 years, treated with secukinumab for moderate-to-severe PsO, or PsA, with at least one retrievable medical examination by hospital medical records were included in this study. Patients were followed for a minimum of 1 month and a maximum of 72 months (8 years). At baseline, 300 mg of the drug was administered weekly during the first 4 weeks and then once monthly. General characteristics were reviewed: sex, age, age of onset, BMI, obesity, cardiovascular comorbidities, diabetes, PsA, naive for biologics, and involvement of difficult sites.

This study was approved by the ethics committee of Turin University hospital (IT10771180014 SS-Dermo20). All patients signed written consent.

### 2.3. Drug Survival

DS was calculated in months on patients at risk of discontinuation and was defined as the time from initiation to discontinuation (stop/switch). The date of discontinuation was defined as the date when the treatment was interrupted due to various reasons such as primary, secondary failure, side effects, or the date when the treatment was switched to another medication for any reason.

### 2.4. Statistical Analysis

For this analysis, epidemiological data (i.e., demographic and disease characteristics and medical history) were summarized using descriptive statistics. Descriptive statistics were used to evaluate the data set according to the number of patients and their percentage proportion in the groups related to the categorical variables; mean and standard deviation (SD) were used for continuous variables. Inferential analyses were performed up to week 260. Categorical variables were analyzed using a chi-square test and Fisher’s exact test as appropriate, while continuous variables were tested using the Shapiro–Wilk test to investigate the normality of the distribution. Dichotomous normal distributions were compared using the student’s *t*-test, and non-normal distributions were tested using the Mann–Whitney U test. The Kruskal–Wallis test was used to compare more than two distributions. Imputation of non-responders was not conducted, and only observed cases were evaluated.

Survival curves were approximated through the Kaplan–Meier estimator and compared using the long-rank test. Proportional hazard Cox regression models were used for multivariate analyses of DS, while both unadjusted and adjusted hazard ratios (HRs) with 95% confidence intervals (CIs) were used to summarize differences. Differences between groups were considered statistically significant for *p* values < 0.05. All statistical analyses were performed with STATA 17 software.

## 3. Results

**Figure 1:**
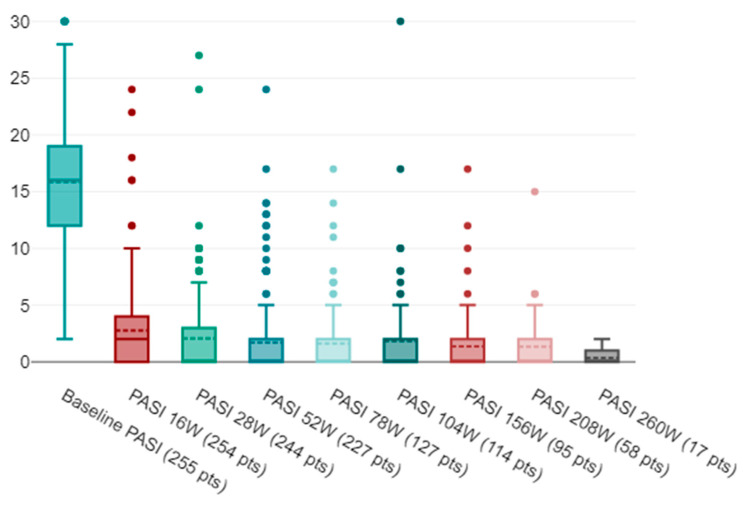
Graphical representation by box and whiskers plot of mean and median reduction in PASI at baseline, at 16, 28, 52, 78, 104, 156, 208, and 260 weeks. On ordinate axis, the absolute pasi, on horizontal axis, the time points; superior and inferior margins of the box represent first and third quartile of the distribution of absolute PASI; the points are the extreme values. PASI, Psoriasis Area Severity Index, W weeks, pts patients.

**Figure 2:**
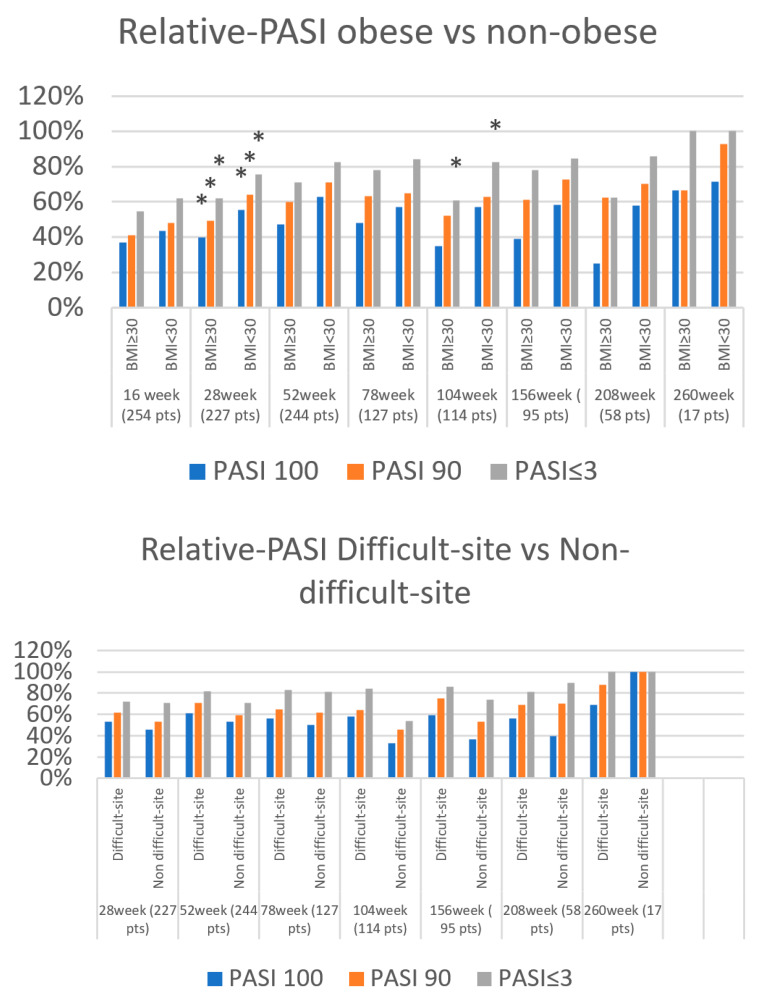

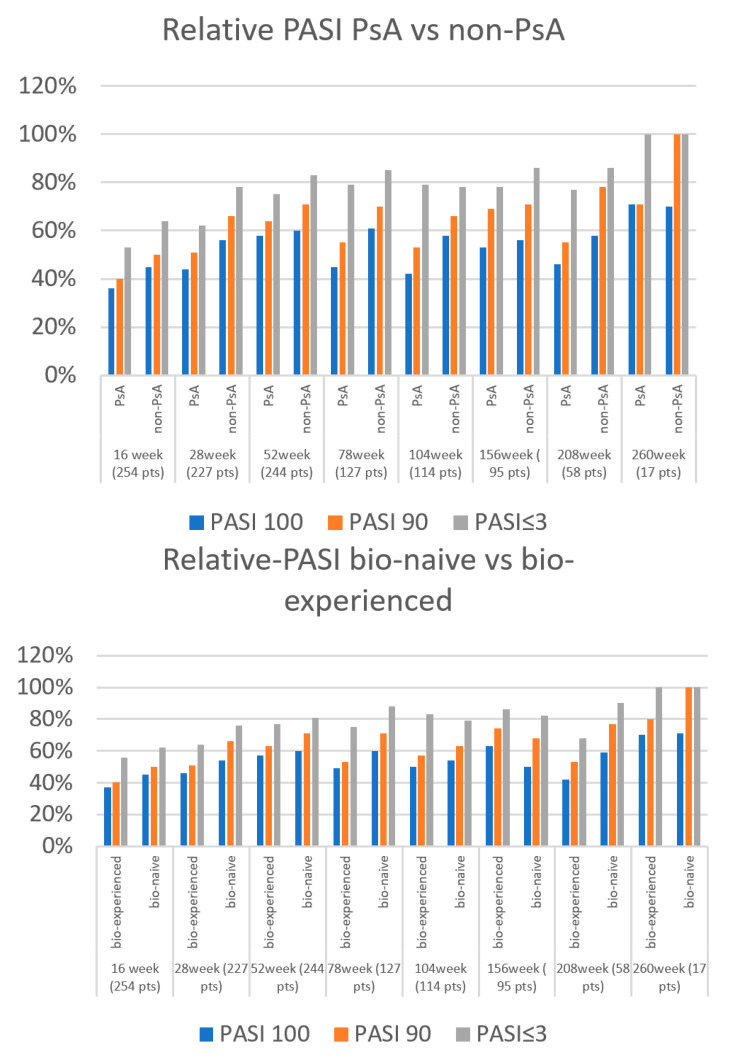

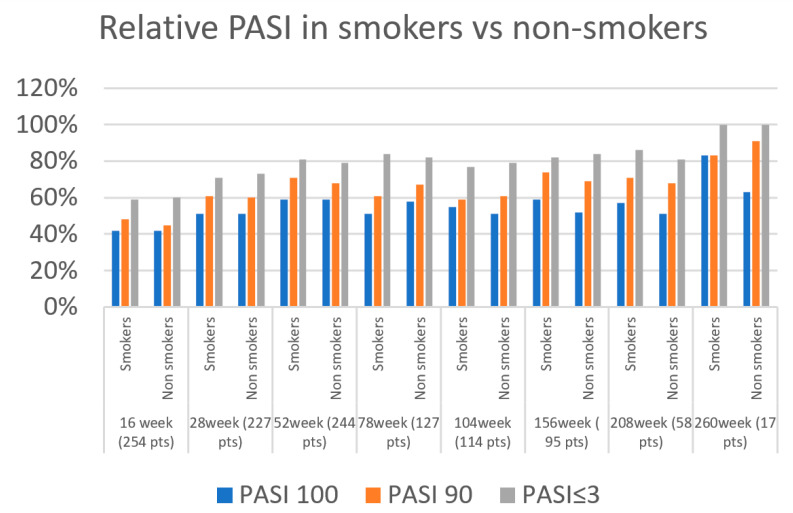
Graphic representation of achievement of PASI 100, PASI90, and PASI < 3 at 16, 28, 52, 78, 104, 156, 208, and 260 weeks, by column chart. Comparison of achievement of PASI 100, PASI90, and PASI < 3 according to obesity, PsA, bio-naïve, difficult-site involvement status, and smoking habits. * Significant *p*-value (<0.05). On ordinate axis, percentage of patients reaching the outcomes, on horizontal axis, the time points according to the dichotomous comparison. The height of the column represents the percentage of patients reaching the outcomes. PASI, Psoriasis Area Severity Index, PsA psoriatic arthritis, pts patients.

In all, 255 patients with moderate-to-severe cutaneous psoriasis, independent of joint involvement, were included in this study (64.3% males and 35.7% females). A total of 254 patients reached follow-up at 16 weeks, followed by 244 at 28 weeks, 227 at 52 weeks, 127 at 78 weeks, 114 at 104 weeks, 95 at 156 weeks, 58 at 208 weeks, and 17 at 260 weeks. The mean age was 58 years (SD 15) and the mean age of onset of psoriasis was 36 years (SD 18.2). Smoking habits were reported by 108 patients (42.35%), PsA was present in 91 patients (35.7%), 27.1% were obese, the mean BMI was 27.6 kg/m^2^ (SD 5.4), 13.4% had diabetes, and 45.6% had cardiovascular comorbidities. Involvement of difficult-to-treat sites was observed in 61 patients (24%). Secukinumab was the first biologic prescribed in 37.7% of patients (Table 1). The mean baseline PASI was 15.8 (SD 5.7) and decreased to 2.8 (SD 3.7) at 16 weeks. This trend was maintained until week 260 with a mean PASI of 0.36 (SD 0.61) (Figure 1). In the observed cases, PASI 100 was reached by 41.7%, 51.2%, 59%, 55.1%, 52.6%, 54.7%, 53.5%, and 70.6% of patients at weeks 16, 28, 52, 78, 104, 156, 208, and 260 (Figure 2). Achievement of PASI 90 showed a similar trend with 46.5% of patients achieving it at week 16, 68.3% at week 52, and 88.2% at week 260 (Figure 2). Absolute PASI ≤ 3 was achieved by 79.7% of patients at week 52 and by all patients at 260 weeks (Figure 2, Table 2).

Considering obesity status, biologic-naive, difficult-site involvement, and PsA, we observed fewer differences in achieving endpoints.

Non-obese patients showed a faster response than obese patients in achieving PASI100, PASI90, and PASI ≤ 3, with significant differences at 28 weeks [55% vs. 40% (*p* = 0.033), 64% vs. 49% (*p* = 0.038), and 76% vs. 62% (*p* = 0.036), respectively] (Figure 2). At the same time points, biological-naïve and absence of joint involvement (PsA) were associated with greater achievement of PASI90 and PASI ≤ 3 (Figure 2). At later time points, a small advantage was observed for non-obese patients in achieving absolute PASI ≤ 3 at 104 weeks (61% vs. 82%, *p* = 0.026) (Figure 2). Patients with involvement of difficult-to-treat sites achieved PASI100 and PASI ≤ 3 at 104 weeks, faster than patients without (58% and 84% vs. 33% and 54% in patients with non-difficult-site involvement, *p* = 0.033 and *p* = 0.001) (Figure 2). At 208 weeks, 77% of bio-experienced vs. 86% of bio-naïve patients achieved PASI ≤ 3 (*p* = 0.044). Smoking habits seemed to not affect relative outcomes (Figure 2) (Table 2).

The estimated DS for secukinumab, analyzed using data from the at-risk patients, was 84.3% at 12 months, 67.8% at 24 months, and 48% at 60 months (Figure 2). The mean follow-up on treatment was 37.3 months (SD 19.7) and 48.2% of patients had interrupted secukinumab at the time of analysis. The mean follow-up before interruption was 21.4 months (SD 19). In all, 50.4% were interrupted due to loss of efficacy, 17.7% due to primary failure, 10.6% for adverse events (two unknown events, two rhinitis, two diarrhea, three candidiasis, one otitis, one transaminitis, and one case of HCV reactivation), one patient was interrupted for the last two reasons, and 20.4% for unknown reasons. Moreover, 27 episodes of side effects were registered: five cases of rhinitis, four cases of asthenia and candidiasis, two cases of headache, diarrhea, and otitis, and one case of nausea, transaminitis, diagnosis of Crohn’s disease, HCV reactivation, arthralgia, and cough.

**Figure 3:**
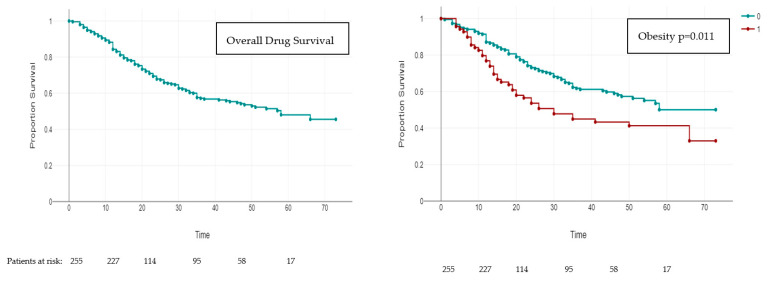

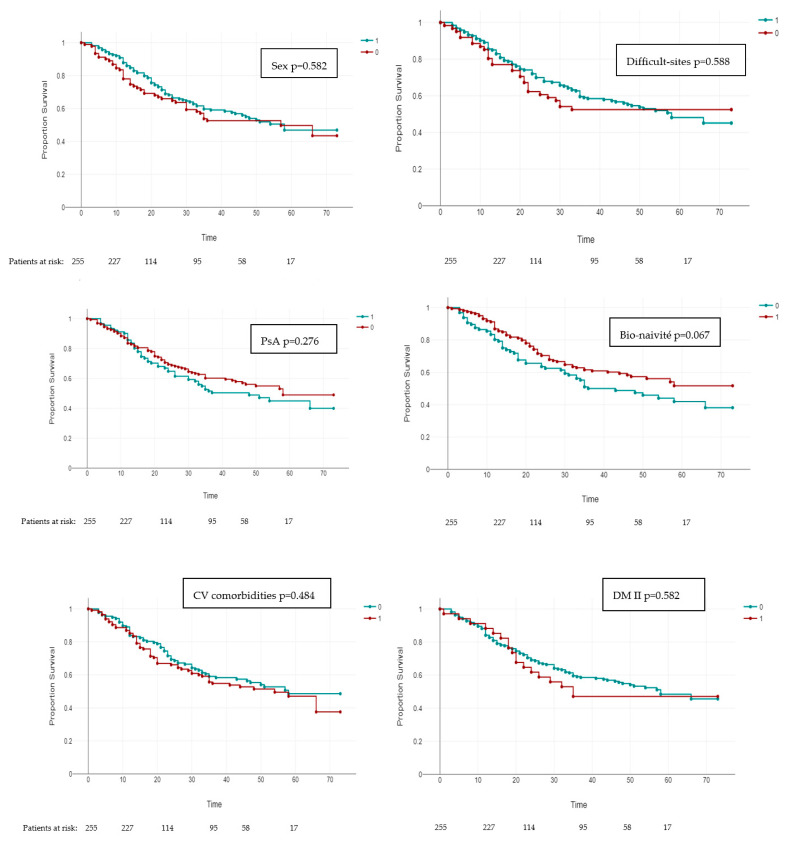

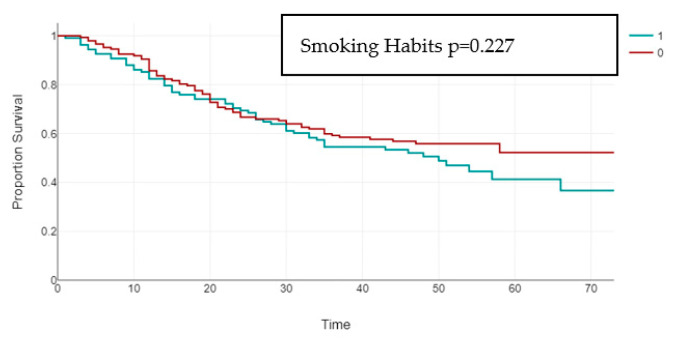
Drug survival in the overall cohort of observed patients and according to sex (1 = male, 0 = female), obesity (1 = presence, 0 = absence), bio-naïve (1 = bio-naïve, 0 = bio-experienced), difficult-site involvement (1 = presence, 0 = absence of difficult site), PsA (1 = presence 0 = absence), CV comorbidities (1 = presence, 0 = absence), T2D (1 = presence, 0 =absence), smoking habits (1 = yes, 2 = no). On ordinate axis, percentage of observed patients still on treatment, on the horizontal axis, time in months. The curve is an estimate of survival according to the number of patients that stop the treatment for any reason. PsA, psoriatic arthritis; CV, cardiovascular; T2D, type II diabetes mellitus.

At univariate analysis, only obesity reduced the DS of secukinumab (*p* = 0.011) with 76% vs. 87% of DS at 52 weeks, 53% vs. 73.1% at 104, and 41.3% vs. 50% at 260 weeks (Figure 3). Obesity and smoking habits were associated with a higher risk of discontinuation in multivariate models (HR 1.6 CI 1.05–2.45, *p* = 0.028; HR 1.48 CI 1.01–2.17, *p* = 0.043, respectively), considering also age, sex, age of onset, difficult-site involvement, PsA, bio-naive, mean baseline PASI, cardiovascular comorbidities, and diabetes (Table 1b).

## 4. Discussion

In our population, secukinumab showed high DS with an estimated 5-year survival in almost half of at-risk patients. The main cause of discontinuation was secondary inefficacy, while adverse events did not frequently lead to treatment discontinuation. Notably, only four cases of candidiasis (1.5%) were recorded during follow-up, which is lower than in other real-world case series where candidiasis ranged from 3–7% of cases [23,24].

Altogether, 84.3% of patients were still on treatment after 1 year of follow-up, which is somewhat lower than reported by Dauden et al. and Wang et al., who found higher survivals of 86–89% [14,21]; Chatzmihail et al., in contrast, reported a low DS of 68.5%, similar to the rheumatologic cohort (PsA and ankylosing spondylitis) of Alonso et al. [20,22]. At two years, DS was 67.8%, which was lower than reported by Mendes-Bastos et al., Ruiz-Villaverde et al., Daudén et al., and in the SERENA study (87%, 71.7%, 74.3%, and 76.4%, respectively) [13,14,25,26]. At 5 years, the survival estimate of 48% is higher than the 4-year survival reported by Nguyen et al. of 34.2%, but lower than the 69.8% reported by Sotiriou et al. [17,18]. Recently, in a large cohort, the RAILWAY study reported a 5-year survival rate of 33.9% in Japanese patients [27].

Obesity and smoking habits are the only two features that negatively predicted treatment response in our population, while bio-naive did not reach statistical significance. Dauén et al. and Sotiriou et al. identified obesity and previous use of other biological therapy as predictors of discontinuation [14,17]. Zhou et al. reported a negative impact on disease control with biologics in patients with smoking habits and a Danish cohort highlighted a specific impact on secukinumab, but other authors reported conflicting results on this topic, and data on treatment survival are few [28,29,30,31]. In contrast, Alonso et al. observed a higher DS in obese patients in a population with PsA and ankylosing spondylitis, identifying only the use of a previous biological drug as a predictor of discontinuation [22]. Ortolan et al. showed a clear difference in DS for patients with PsA at 12 and 24 months up to 20 percentage points; in our case series, a substantial overlap was observed between the two populations with 85% vs. 83% at 12 months and 69% vs. 68% at 24 months in patients without and with joint involvement, respectively [16]. Similar to the study by Sotiriou et al., no differences were observed in DS with respect to difficult-to-treat sites, although the Portuguese cohort had lower retention rates in the case of involvement of these sites [13,17].

Good effectiveness was observed at multiple time points in our population, achieving PASI100, PASI90, and PASI < 3 in 55.1%, 68.3%, and 79.7% of patients at 52 weeks, respectively, and 70.6%, 88.2%, and 100% at 260 weeks, respectively. Our 1-year percentage is lower than that of Sotiriou et al. for PASI100 and PASI90 (63.8% and 71.3%, respectively), but higher at 5 years even if the Greek case series had more patients (57.6% and 68.1%) [17]. Our observations are in line with what was reported by Nguyen et al. at 4 years for PASI90 and PASI100 (78% and 52% vs. 69% and 53.5%, respectively, in Nguyen’s case series and ours) [18]. In general, in our population, complete response rates to treatment were higher than those reported in other real-life experiences at 1 and 2 years, and compared well to the long-term extension of the phase III ERASURE and FIXURE studies with PASI100 reported in 35.1% of patients [8,19,21].

Obesity was the main unfavorable factor of DS with lower rates of achievement of endpoints at week 28. More infrequently, disadvantages were observed for bio-experienced and PSA patients at several time points. Surprisingly, at 104 weeks patients with involvement of difficult sites experienced better responses than those without involvement. In general, with longer follow-up, the differences in the four groups decreased, demonstrating a progressive achievement of secukinumab targets in patients continuing therapy.

Curiously, joint involvement in our population would not seem to result in a higher rate of therapeutic discontinuation. In the few real-life studies addressing the topic of psoriatic arthritis and axial PsA, secukinumab was effective and safe in controlling disease outcomes (such as DAPSA and MDA), with survival ranging from 40% to 70% at 2 years depending on the number of prior anti-TNFAalpha-based lines of therapy [22,32].

Regarding safety, secukinumab did not record any serious adverse events. Only one patient was diagnosed with Chrohn’s disease during treatment, but in a mild form. It is worth mentioning that the occurrence of Inflammatory bowel disease (IBD) can be triggered by IL-17 inhibitor therapy in patients with PsO and especially PsA. Adequate patient selection is mandated before the initiation of secukinumab, as the development of IBD resistant to approved treatment with IL17A interleukin inhibition is common [9,33,34].

It is the opinion of the author that it should be mandated to implement all possible strategies to increase the survival of a biological treatment even considering the cost of a therapeutic failure. Apart from adverse events, in patients who begin to show signs of loss of efficacy, it seems appropriate to favor clearance by topical treatment or the possible addition of systemic drugs such as methotrexate and acitretin. Alternatively, pharmacological dose escalation, although related to increased costs, seems an appropriate strategy to be applied for a short time in patients with a progressive loss of response with secukinumab [35].

The real-life and retrospective nature of this study is its main limitation. The analysis of observed cases limits the observation of effectiveness to patients who continued treatment by not imputing treatment interruptions, and the low sample size at 206 and 260 weeks reduces the generalizability of our observations. Regarding PsA, the absence of registered outcomes for joint domains is another relevant limitation.

## 5. Conclusions

Secukinumab appears to be effective and safe for up to 5 years of treatment of moderate–severe psoriasis, with a high DS at later time points. Obesity appears to be the main obstacle for response and maintenance of DS. Difficult-to-treat site involvement, previous use of biological drugs, and joint involvement had little impact on the long-term response and therapeutic survival of secukinumab.

## Figures and Tables

**Table 1:** (**a**) General characteristics of observed patients at baseline; (**b**) Proportional hazard Cox regression models for predictors of drug interruption (titled: predictors of secukinumab interruption). Age, sex, smoking habits, age of onset, difficult-site involvement, PsA, bio-naive, mean baseline PASI, obesity, CV. M, male; BMI, body mass index; PsA, psoriatic arthritis; CV, cardiovascular; T2D, type II diabetes mellitus; PASI, Psoriasis Area Severity Index; HR hazard ratio.

(a) General Characteristics of the Patients				
	Patients (n = 255)			
**Sex (M) %**	**64.3%**			
Mean age (years)	58 (SD 15)			
Mean age of onset (years)	36 (SD 18.2)			
BMI (kg/m^2^) (mean)	27.6 (SD 5.4)			
Smoking Habits (yes)	42.35%			
PsA %	35.7%			
Obesity (BMI ≥ 30)%	27.1%			
CV comorbidities %	45.6%			
T2D	13.4%			
Bio-Naive	37.7%			
Difficult-site Involvement	24%			
PASI baseline (mean)	15.8 (SD 5.7)			
**(b) Predictors of Secukinumab Interruption**				
	*p*	HR	Lower 95% CI	Upper 95% CI
Age (years)	0.19	1.01	0.99	1.03
Sex (male)	0.605	0.91	0.62	1.32
Smoking Habits (yes)	0.043	1.48	1.01	2.17
Age of onset (years)	0.078	0.99	0.97	1
Difficult-site involvement	0.365	0.82	0.54	1.26
PsA	0.81	1.05	0.71	1.56
Bio-naive	0.199	0.77	0.53	1.14
Mean baseline PASI	0.8	1	0.96	1.03
Obesity (BMI ≥ 30)	0.028	1.61	0.05	2.45
CV comorbidities	0.993	1	0.64	1.56
T2D	0.957	00.98	0.54	1.79

**Table 2:** Percentage achievement of PASI 100, PASI90, and PASI < 3 at 16, 28, 52, 78, 104, 156, 208, and 260 weeks according to obese, PsA, bio-naïve, and difficult-site involvement status. In bold, significant *p*-value (<0.05). PASI, Psoriasis Area Severity Index; BMI, body mass index kg/m^2^, PSA, psoriatic arthritis.

	BMI ≥ 30	BMI < 30	*p*	PSA	NON PSA	*p*	Bio-Exp	Bio-Naive	*p*	Difficult-Site	Non-Difficult-Site	*p*	Smokers	Non-Smokers	*p*
**PASI 100 16 W N°, %**	25/68, 36.8%	81/186, 43.5%	0.332	33/91, 36.3%	73/163, 44.8%	0.187	35/95, 36.8%	71/159, 44.7%	0.222	79/193, 40.9%	26/60, 43.3%	0.742	45/108, 41.7%	61/146, 41.8%	0.985
**PASI 90 16 W N°, %**	28/68, 41.1%	90/186, 48.4%	0.308	36/91, 39.6%	82/163, 50.3%	0.1	38/95, 40%	80/159, 50.3%	0.111	88/193, 45.6%	29/60. 48.3%	0.71	52/108, 48.1%	66/146, 45.2%	0.642
**PASI ≤ 3 16 W N°, %**	37/68, 54.4%	115/186, 61.8%	0.286	48/91, 52.7%	104/163, 63.8%	0.085	53/95, 55.8%	99/159, 62.3%	0.308	116/193, 60.1%	35/60, 58.3%	0.807	64/108, 59.3%	88/146, 60.3%	0.87
**PASI 100 28 W N°, %**	25/63, 39.7%	100/181, 55.2%	**0.033**	39/89, 43.8%	86/155, 55.5%	0.079	40/87, 46%	85/157, 54%	0.222	99/188, 52.7%	25/55, 45.5%	0.347	53/104, 51%	72/140, 51.4%	0.942
**PASI 90 28 W N°, %**	31/63, 49.2%	116/181, 64.1%	**0.038**	45/89, 50.6%	102/155, 65.8%	**0.019**	44/87, 50.6%	103/157, 65.6%	**0.022**	117/188, 62.2%	29/55, 52.7%	0.205	63/104, 60.6%	84/140, 60%	0.927
**PASI ≤ 3 28 W N°, %**	39/63, 61.9%	137/181, 75.5%	**0.036**	55/89, 61.8%	121/155, 78.1%	**0.006**	56/87, 64.4%	120/157, 76.4%	**0.044**	136/188, 72.3%	39/55, 70.9%	0.835	74/104, 71.2%	102/140, 72.9%	0.769
**PASI 100 52 W N°, %**	26/55, 47.3%	108/172, 62.8%	**0.042**	48/83, 57.8%	86/144, 59.7%	0.78	45/79, 57%	89/148, 60.1%	0.643	106/175, 60.6%	27/51, 52.9%	0.33	57/96, 59.4%	77/131, 58.8%	0.928
**PASI 90 52 N°, % W**	33/55, 60%	122/172, 70.9%	0.129	53/83, 63.9%	102/144, 70.8%	0.277	50/79, 63.3%	105/148, 70.9%	0.238	124/175, 70.9%	30/51, 58.8%	0.105	66/96, 71%	89/131, 67.9%	0.897
**PASI ≤ 3 52 W N°, %**	39/55, 70.9%	142/172, 82.6%	0.061	62/83, 74.7%	119/144, 82.6%	0.152	61/79, 77.2%	120/148, 81.1%	0.49	144/175, 82.3%	36/51, 70.6%	0.068	78/96, 81.3%	103/131, 78.6%	0.627
**PASI 100 78 W N°, %**	13/27, 48.1%	57/100, 57%	0.412	21/47, 44.7%	49/80, 61.3%	0.07	22/47, 46.8%	48/80, 60%	0.149	57/101, 56.4%	13/26, 50%	0.556	25/49, 51%	45/78, 57.7%	0.462
**PASI 90 78 W N°, %**	17/27, 63%	65/100, 65%	0.359	26/47, 55.3%	56/80, 70%	0.095	25/47, 53.2%	57/80, 71.3%	**0.04**	66/101, 65.3%	16/26, 61.5%	0.717	30/49, 61.2%	52/78, 66.7%	0.533
**PASI ≤ 3 78 W N°, %**	21/27, 77.8%	84/100, 84%	0.448	37/47, 78.7%	68/80, 85%	0.367	35/47, 74.5%	70/80, 87.5%	0.061	84/101, 83.2%	21/26, 80.8%	0.773	41/49, 83.7%	64/78, 82.1%	0.814
**PASI 100 104 W N°, %**	8/23, 34.8%	52/91, 57.1%	0.055	16/38, 42.1%	44/76, 57.9%	0.111	21/42, 50%	39/72, 54.2%	0.667	52/90, 57.8%	8/24, 33.3%	**0.033**	24/44, 54.5%	36/70, 51.4%	0.746
**PASI 90 104 W N°, %**	12/23, 52.2%	57/91, 62.6%	0.359	20/38, 52.6%	49/76, 64.5%	0.223	24/42, 57.1%	45/72, 62.5%	0.572	58/90, 64.4%	11/24, 45.8%	0.097	26/44, 59.1%	43/70, 61.4%	0.804
**PASI <3 104 W N°, %**	14/23, 60.9%	75/91, 82.4%	**0.026**	30/38, 78.9%	59/76, 77.6%	0.873	32/42, 83.3%	57/72, 79.2%	0.711	76/90, 84.4%	13/24, 54.2%	**0.001**	**34/44, 77.3%**	**55/70, 78.6%**	**0.87**
**PASI 100 156 W N°, %**	7/18, 38.9%	45/77, 58.4%	0.134	19/36, 52.7%	33/59, 55.9%	0.764	22/35, 62.9%	30/60, 50%	0.225	45/76, 59.2%	7/19, 36.8%	0.08	20/34, 58.8%	32/61, 52.5%	0.55
**PASI 90 156 W N°, %**	11/18, 61.1%	56/77, 72.7%	0.33	25/36, 69.4%	42/59, 71.1%	0.857	26/35, 74.3%	41/60, 68.3%	0.539	57/76, 75%	10/19, 52.6%	0.056	25/34, 73.5%	42/61, 68.9%	0.632
**PASI <3 156 W N°, %**	14/18, 77.8%	65/77, 84.4%	0.498	28/36, 77.8%	51/59, 86.4%	0.274	30/35, 85.7%	49/60, 81.7%	0.611	65/76, 85.5%	14/19, 73.7%	0.217	28/34, 82.4%	51/61, 83.6%	0.876
**PASI 100 208 W N°, %**	2/8, 25%	29/50, 58%	0.082	10/22, 45.5%	21/36, 58.3%	0.34	8/19, 42.1%	23/39, 59%	0.227	27/48, 56.3%	4/10, 40%	0.349	12/21, 57.1%	19/37, 51.4%	0.671
**PASI 90 208 W N°, %**	5/8, 62.5%	35/50, 70%	0.67	12/22, 54.5%	28/36, 77.8%	0.064	10/19, 52.6%	30/39, 76.9%	0.061	33/48, 68.8%	7/10, 70%	0.938	15/21, 71.4%	25/37, 67.6%	0.76
**PASI <3 208 W N°, %**	5/8, 62.5%	43/50, 86%	0.102	17/22, 77.3%	31/36, 86.1%	0.387	13/19, 68.4%	35/39, 89.7%	**0.044**	39/48, 81.3%	9/10, 90%	0.505	18/21, 85.7%	30/37, 81.1%	0.227
**PASI 100 260 W N°, %**	2/3, 66.6%	10/14, 71.4%	0.87	5/7, 71.4%	7/10, 70%	0.1	7/10, 70%	5/7, 71.4%	0.949	11/16, 68.8%	1/1, 100%	0.506	5/6, 83.3%	7/11, 63.6	0.6
**PASI 90 260 W N°, %**	2/3, 66.6%	13/14, 92.9%	0.201	5/7, 71.4%	10/10, 100%	0.072	8/10, 80%	7/7, 100%	0.208	14/16, 87.5%	1/1, 100%	0.707	5/6, 83.3%	10/11, 90.9%	1
**PASI <3 260 W N°, %**	3/3, 100%	14/14, 100%	1	7/7, 100%	10/10, 100%	1	10/10, 100%	7/7, 100%	1	16/16, 100%	1/1, 100%	1	6/6 100%	11/11 100%	1

## Data Availability

Data available upon request.
